# Validation and investigation of cross cultural equivalence of the Fremantle back awareness questionnaire - German version (FreBAQ-G)

**DOI:** 10.1186/s12891-021-04156-1

**Published:** 2021-04-02

**Authors:** Axel Schäfer, Benedict M. Wand, Kerstin Lüdtke, Katja Ehrenbrusthoff, Thomas Schöttker-Königer

**Affiliations:** 1 Faculty of Social Work and Health, University of Applied Sciences and Arts (HAWK), Hildesheim, Germany; 2 School of Physiotherapy, University of Notre Dame Australia, Fremantle, Australia; 3 University of Luebeck, Medical Section, Orthopaedics and Trauma Surgery, Academic Physiotherapy, Pain and Exercise Research (PERL), Luebeck, Germany; 4 Department of Applied Health Sciences, Hochschule für Gesundheit, Bochum, Germany

**Keywords:** Low back pain, Disrupted self-perception, Body image, Item response theory, Validity

## Abstract

**Background:**

Disrupted self-perception of the low back might contribute to chronic non-specific low back pain. The Fremantle back awareness questionnaire is a simple questionnaire to assess back specific self-perception. The questionnaire has recently been translated to German (FreBAQ-G). The aim was to further investigate the psychometric properties of the FreBAQ-G, to evaluate its cross cultural validity in patients with chronic non-specific LBP and to explore potential relationships between body perception, pain, disability and back pain beliefs.

**Methods:**

In this cross-sectional multicentre study, sample data were merged with data from the validation sample of the original English version to examine cross-cultural validity. Item Response Theory was used to explore psychometric properties and differential item function (DIF) to evaluate cross-cultural validity and item invariance. Correlations and multiple linear regression analyses were used to explore the relationship between altered back specific self- perception and back pain parameters.

**Results:**

Two hundred seventy-two people with chronic low back pain completed the questionnaires. The FreBAQ-G showed good internal consistency (Cronbach’s alpha = 0.84), good overall reliability (r = 0.84) and weak to moderate scalability (Loevinger Hj between 0.34 and 0.48). The questionnaire showed unidimensional properties with factor loadings between 0.57 and 0.80 and at least moderate correlations (r > 0.35) with pain intensity, pain related disability and fear avoidance beliefs (FABQ total - and subscores). Item and test properties of the FreBAQ-G are given. Only item 7 showed uniform DIF indicating acceptable cross-cultural validity.

**Conclusions:**

Our results indicate that the FreBAQ-G is a suitable questionnaire to measure back specific self-perception, and has comparable properties to the English-language version.

**Supplementary Information:**

The online version contains supplementary material available at 10.1186/s12891-021-04156-1.

## Background

Low back pain (LBP) is the leading cause for years lived with disability worldwide [1], thus remaining the “medical catastrophe” described by Waddell back in 1998 [2]. Increasing prevalence and steadily rising costs not only occur in western industrialised countries, but also increasingly in mid- to lower income countries [1]. Prevalence rates in Germany are particularly high, with a point prevalence of 34.2% (95% CI 33.2–35.1%) and a lifetime prevalence of 85.2% (95% CI, 84.4–85.9%) [3]. In Germany, back pain is responsible for approximately every 10th day of work absence [4]. Worldwide, point prevalence was estimated to be 18.3% (SD 11.7%) and lifetime prevalence 38.9% (SD 24.3%) [5].

While there is consensus that non-specific LBP is a multifaceted health problem with complex interactions between various biological, social and psychological factors [6], the challenge remains to identify causative characteristics in order to develop effective targeted treatment strategies. Evidence suggests that changes in the way the physical body is represented within the central nervous system and associated changes in the way the body part is perceived and experienced contribute to chronic pain states such as phantom limb pain, complex regional pain syndrome and LBP [7–9]. Self-perception, defined here as how the body feels to the person [10], is formulated by a complex interplay of the information coded within the central nervous system that represent the body’s shape and size, ongoing sensory and motor information, as well as thoughts and beliefs about the body [10]. Self-perception has received considerable attention in the pain literature. For example, people with LBP have been asked to complete drawings of how they perceive their back, and commonly represent the back as distorted or report difficulty perceiving the outline of the back [11, 12]. In addition, individual mechanisms thought to contribute to self-perception such as tactile [13] and proprioceptive [14] acuity may also be impaired in people with LBP. Furthermore, preliminary evidence suggests that treatment programmes addressing these issues may improve pain and function in LBP [15, 16]. More recent data suggests that body representation problems in those with back pain may extend beyond the perpetual and also encompass the cognitive-affective dimension of body image such as self-acceptance, physical efficacy and body satisfaction [17, 18].

To assess back specific self-perception in persons with LBP, the Fremantle Back Awareness Questionnaire (FreBAQ) was recently developed [19] and validated [20]. Meanwhile, the FreBAQ was translated to German following international guidelines for the transcultural adaptation of self-reported measures [21]. The first step of cross cultural adaptation, the translation process and evaluation of reliability and known groups validity of the translated questionnaire is described in detail elsewhere [22]. The German version (FreBAQ-G) demonstrated moderate inter- and intratester reliability and known-groups validity [22]. The final stages of cross-cultural adaptation including cross cultural validity and equivalence of item and score properties as recommended by Mokkink et al. [23] have not been investigated yet. The aim of this article is to present the outcomes of a further, more comprehensive evaluation of the FreBAQ-G using Item Response Theory (IRT) in a large sample of persons with non-specific chronic low back pain (NSCLBP) and to investigate cross cultural validity / measurement invariance as well as item and score properties of the FreBAQ-G.

## Methods

### Research questions

#### The study had the following objectives

1. To investigate the psychometric properties of the FreBAQ-G using Item Response Theory. Based on IRT modelling item functioning characteristics, such as item difficulty, discrimination and item information were examined. In addition the distribution of the items over the scale as well as test-score properties including reliability parameters and measurement error were evaluated. Finally item invariance of the FreBAQ-G was assessed to examine whether the questionnaire behaves in the same way in different subgroups of the German speaking population.
2. To investigate cross-cultural validity / item invariance of the FreBAQ-G in patients with NSCLBP. Based on IRT techniques differential item functioning (DIF) was used to evaluate whether the translated version behaves in the same way in the German speaking population as the original version in the English speaking population.
3. To investigate hypothesis based construct validity of the FreBAQ-G by evaluation of the correlations of back specific self-perception with other back pain related parameters such as pain intensity, function and fear avoidance beliefs corresponding to the English validation study [20].

### Study design

The study was designed as a multicentre, cross-sectional study. Data was collected as part of a study evaluating lumbar movement control in persons with NSCLBP. All participants provided written informed consent and all procedures conformed to the Declaration of Helsinki. To investigate cross-cultural validity the data of this study were pooled with those collected by Wand et al. [20] .

### Setting

Participants were recruited in seven physiotherapy practices in Germany between April and September 2019.

### Participants

Participants had to meet the following inclusion criteria: age ≥ 18 years; sufficient German language ability to complete the questionnaire; currently experiencing NSCLBP with or without leg pain (leg pain above the knee and main pain had to be localized below the costal margin and above the inferior gluteal folds) and duration of symptoms ≥3 months. The pain level, calculated as the mean of the actual pain intensity and the average pain intensity during the last 3 months, measured on an 11-point numeric rating scale (NRS), needed to be above 0. Participants were excluded if they had signs and symptoms indicating specific spinal pathology [24].

### Bias

Data collection and data analysis was conducted by different persons to minimize potential risk of bias.

### Procedure

Participants provided basic demographic information and completed a self-developed questionnaire to collect information about LBP characteristics. Pain related disability during daily activities, leisure time and work, as well as pain intensity, were assessed using 11-point numerical rating scales (NRS 0 = no pain / disability - 10 = worst imaginable pain / disability). For overall pain related disability we calculated the mean of the impairment scores during daily activities, leisure time and work. Pain related fear was estimated using the German version of the Fear Avoidance Beliefs Questionnaire (FABQ) [25]. Finally, the participants completed the FreBAQ-G [22]. The FreBAQ-G consists of nine items measuring back specific self-perception on a five point rating scale with a range 0–36 (higher values indicating greater levels of impairment).

### Sample size

For questionnaires with ordinal scaled items, polytomous item response models are recommended [26]. The COSMIN (Consensus-based Standards for the selection of health status measurement instruments) checklist advocates a minimum sample size of 200 participants for IRT based Rasch analyses [23]. However, for polytomous IRT models the sample size should be at least 250, but 500 for accurate parameter estimates is recommended [27]. To assess the psychometric properties of the German Version we aimed to recruit a sample greater than 250. To evaluate cross cultural validity we pooled our German data set with the English-language data set collected by Wand et al. [20]. The sample size of the English data set consists of 251 participants with NSCLBP. So the overall sample size to investigate cross cultural validity meets the recommendation of 500 participants.

### Data analysis

#### Descriptive statistics

Descriptive statistics were used to describe the demographic and clinical characteristics of the sample. The FreBAQ-G was summarized using range, median, mean and standard deviation for the total score. The frequencies in each response category were also reported.

IRT modelling was used to assess cross cultural validity and the psychometric properties of the FreBAQ-G. Because the 9 items of the FreBAQ-G are ordinal scaled, a polytomous IRT model, should be used [26]. Based on statistical analysis the graded response model (GRM) was selected [26]. The assumptions of the statistical IRT model, local independence, dimensionality and model fit statistics were investigated. Details about the model selection and test of the IRT assumptions are given in the Appendix.

#### Psychometric properties of the FreBAQ-G

Psychometric properties, including scalability, internal consistency, item characteristics, test characteristics and test reliability of the FreBAQ-G were calculated. Differential item functioning (DIF) was used to evaluate item invariance, which means whether different subgroups of the German speaking sample have the same chance to answer the items of the FreBAQ-G.

Internal consistency was estimated using Cronbach’s α. Acceptable internal consistency is reached if α is > 0.7 [28]. Loevinger’s H_j_ scalability coefficient is reported as a measure of homogeneity. The coefficient can be considered as an accuracy measure for the ability of items to order the respondents in the measured latent trait (back specific self-percetion) [29]. As a rule of thumb, items with values of Loevinger’s H_j_ < 0.3 are indicative of poor/no scalability, values between 0.3 and 0.4 indicate useful but weak scalability, values between 0.4 and 0.5 are indicative of moderate scalability and values > 0.5 indicate strong scalability [30].

After fitting the GRM model, the test- and item-characteristics were evaluated. In IRT modelling, a person’s ability in the latent trait -in this study” back specific self-perception”- is measured on a logit scale which follows a Z-distribution with a mean of 0 and a SD of 1 (range from − 4 to 4) [26]. This logit scale is called Theta (θ) and is represented on the x-axis of every IRT graph. The θ -scale is not sample specific [26, 31, 32], so that even when the questionnaire is administered to other groups or languages, the items should have the same properties, yielding comparable scores. Hence, the item and test characteristics of the current study should be comparable to those of the original English speaking version reported by Wand et al. [20].

The test characteristic curve visualizes the relationship between the IRT-based estimated ability in the latent trait “back specific self-perception” for each person and the expected classical sum-score, based on the classical test theory [26]. This helps to understand which FreBAQ-G sum-score is expected for a person with NSCLBP with a certain trait level on the current scoring system.

The test information function shows how precisely the FreBAQ-G can estimate the level of the respondent’s ability in the latent trait [26]. Thereby, the test information function helps to decide which region on the latent trait continuum can be estimated most precisely (or most poorly). This concept is closely related to the concept of reliability [32], therefore the test information function also visualizes the standard error (SE). In IRT, the SE varies for each level of the latent trait. The SE can be used to calculate the estimated overall mean reliability often described as marginal reliability, using the formula: reliability = 1-mean (SE)^2^ [33].

The item characteristics include item discrimination (slope), item difficulty (threshold) and item information [26]. The item discrimination parameter (a) describes the slope of the item characteristic curve. Higher values are indicating better item discrimination, which means items with higher values are more sensitive to detect a difference in the latent trait (back specific self-perception). Values > 1 are desirable [26]. Item discrimination and item information are very closely related [26, 32]. The item difficulty parameter (b) describes the point on the x-axis (θ value), where the probability of choosing a response option is 50% (threshold). Because of the underlying statistical nature of the GR model the item difficulty parameters are cumulative [26]. Item difficulty parameters are calculated for each item. A person whose back specific self-perception is not impaired will choose the response option 0 (never feels like that), whereas a person with highly impaired back specific self-perception should have a high probability to choose response option 4 (always, or most of the time feels like that). The highest probability of which response option will be answered by a person with a certain trait level is visualized in the category characteristic curve.

Finally, differential item function (DIF) was used to assess the assumption of item invariance [26]. Item invariance implies that the FreBAQ-G is independent to particular sample characteristics. Differential item function (DIF) is present for a given item if individuals with the same ability level (back specific self-perception), but belonging to different groups (e. g. gender), do not have the same probability (chance) of responding to the item with the same rating [26]. Therefore, item invariance can be considered as a measure of fairness.

#### Cross cultural validity

Cross cultural validity refers to the equivalence of measurement across different cultural groups [28]. Cross cultural validity was investigated using IRT techniques. In a first step we pooled the data of the German version (FreBAQ-G, *N* = 271) with those collected for the English-language validation study (FreBAQ, *N* = 251) in an Australian study population [20]. To detect differential item function (DIF) we first separately investigated the item properties (difficulty and discrimination) for the German and English version using graded response model (GRM). To differentiate between uniform (difference in item difficulty only) and non-uniform (difference in item difficulty and discrimination) differential item function (DIF), the mean item difficulty was calculated per polytomous item when the slopes over all items were set to 1 [34]. The calibrated mean item difficulties were plotted with the German items on the y-axis and the English items on the x-axis. To facilitate interpretation an identity line was drawn through the origin of the plot with a slope of 1. Additionally control lines representing 95% CI are drawn around the identity line. Items that fall outside these control lines are suspected to demonstrate differential item function (DIF) [28, 31]. In the same way the item discrimination parameters were plotted. In addition we used the IRT-LR test (likelihood ratio test) to confirm both uniform and non-uniform differential item function (DIF) [34, 35]. The IRT-LR test procedure compares hierarchically nested IRT models; with one model fully constraining the IRT parameters to be equal between the German and the English version and other models that allows the item parameters to be freely estimated between groups. Finally we used a multiple-group graded response model (GRM) model with a correction for observed differential item function (DIF) to validate the performance of the classical sum-score of the English and German version [34].

#### Construct validity: associations of self-perception of the back with back pain related parameters

The relationship between the IRT-based estimated FreBAQ-G score (Theta) and pain intensity, disability and fear avoidance beliefs was calculated using correlation statistics (Pearsons r coefficient). Finally multiple linear regression with the FreBAQ-G (estimated with the Theta) as the dependent variable was performed to find the best predictors.

For statistical analyses Stata 16.1 (StataCorp LLC, USA) was used. The IRT model fit statistics was calculated using the student version of IRTPRO 4.2 (Scientific Software International Inc., USA).

## Results

### Participant characteristics

Two hundred seventy-two patients with NSCLBP were included of which 271 completed all questionnaires. Table 1 gives a summary of the demographic data of this sample and of the sample used by Wand et al. [20] for the validation of the original English version of the FreBAQ.

**Table 1:** Demographic data of participants of this study and the English language population collected by Wand et al. [20]

	German language sample (this study)	English language sample
Sample size	*n* = 272	n = 251
Age (years) mean (SD)	42.4 (±15.8)	48.8 (±13.4)
Height (cm) mean (SD)	173.8 (±9.4)	170.9 (±9.8)
Weight (kg) mean (SD)	75 (±16)	80.6 (±16.7)
Body mass index (BMI), mean (SD)	24.75 (±4.4)	27.6 (±5.2)
Sex female n (%)	167 (61.4)	148 (59.0)
Average back pain intensity (SD)	3.8 (±2)	5.8 (±1.9)
Fear avoidance (FABQ-PA scale) (SD)	9.6 (±5.8)	14.1 (±6)
FreBAQ (SD)	8.0 (±6)	9.8 (±6.6)

*SD* Standarddeviation, *FABQ-PA scale* Fear avoidance beliefs questionnaire-subscale physical activity, *FreBAQ* Freemantle back awareness questionaire

At the time of measurement, the average pain intensity within the last 3 months was 3.8 (SD 2) and the actual pain intensity was 3.4 (SD 2.1) measured on a 0–10 NRS. The average pain level was 3.6 (SD 1.7) and the average pain related disability was 3 (SD 2.1) on a 0–10 NRS.

The mean Fear Avoidance Beliefs Questionnaire (FABQ) score of all participants (*N* = 272) was 20.4 (SD = 12) (range 0–60, higher values indicating greater levels of fear avoidance). The mean value in the subscale physical activity (FABQ-PA) was 9.6 (SD 5.8) (range 0–24) and 10.8 (SD 9) in the work subscale (FABQ-W; range 0–42). At the time of measurement, 99 participants were receiving physiotherapy treatment.

**Fig. 1:**
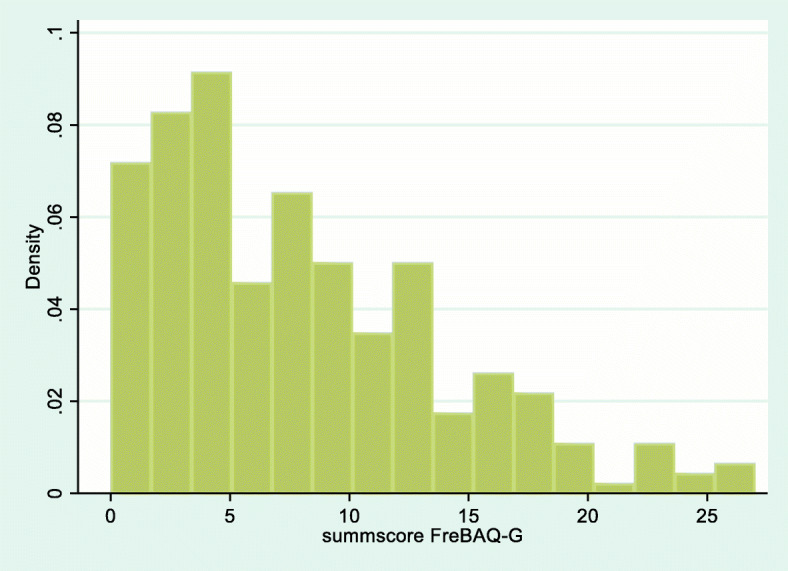
Sumscore FreBAQ Range 0–36, higher values indicate greater levels of impaired back specific self-perception

The average total FreBAQ-G score was 8.0 (SD 6, range 0–27), with a median of 7.0 (interquartile range 3–12). Figure 1 shows the distribution of the FreBAQ-G sum-scores.

Two hundred sixty-four subjects had no missing values, 6 subjects had 1 missing value each, 1 subject had 5 missing values and 1 subject had 9 missing values (see also Table 2).

**Table 2:** Frequencies and responses to each FreBAQ-G item

Item	Miss-ing %	N	response categories in %							Alpha item	Loev Hj coeff
			Never feels like that (0)	Rarely feels like that (1)	Occ. or some of the times feel like that (2)	Often, or a moderate amount of time feels like that (3)	Always, or most of the time feels like that (4)	Median	Mean		
1	0.74	270	49,89	24,44	19,26	6,67	0.74	1	0.85	0.82	0.43
2	1.10	269	28,62	29,37	25,65	13,38	2,97	1	1.3	0.83	0.42
3	0.74	270	47,41	31,11	15,56	5,56	0.37	1	0.79	0.82	0.45
4	0.74	270	41,85	28,15	17,04	11,48	1,48	1	1.01	0.82	0.48
5	1.47	268	43,28	25,37	17,16	11,19	2,99	1	1.04	0.82	0.47
6	1.10	269	60.97	23,42	7,43	6,32	1,86	0	0.64	0.82	0.45
7	0.37	271	71,22	14,76	9,59	4,43	–	0	0.47	0.83	0.39
8	0.74	270	79,63	12,59	5,93	1,85	–	0	0.29	0.84	0.39
9	0.37	271	25,46	22,14	26,20	20,30	5,90	2	1.57	0.84	0.34
Scale			Cronbach alpha (interrelatedness) 0.84								

### Psychometrics of the FreBAQ-G

The frequencies, median responses and missing values to each FreBAQ-G item are given in Table 2. Response option 4 (always or most of the time feels like that) was not chosen for items 7 and 8. For items 1 to 8, the distribution of the responses to each category is left skewed towards the option 0 (never feels like that), whereas for item 9 the responses are more equally distributed. Loevinger Hj coefficients are between 0.34 and 0.48 (homogeneity of the scale: 0 represents no correlation and 1 represents a perfect Guttmann scale), with the lowest value for item 9 and the highest value for item 4. Cronbach’s alpha for the scale is 0.84 (test score reliability coefficient: 0 represents no interitem correlation 1 represents perfect interitem correlation).

### Test characteristics of the FreBAQ-G

**Fig. 2:**
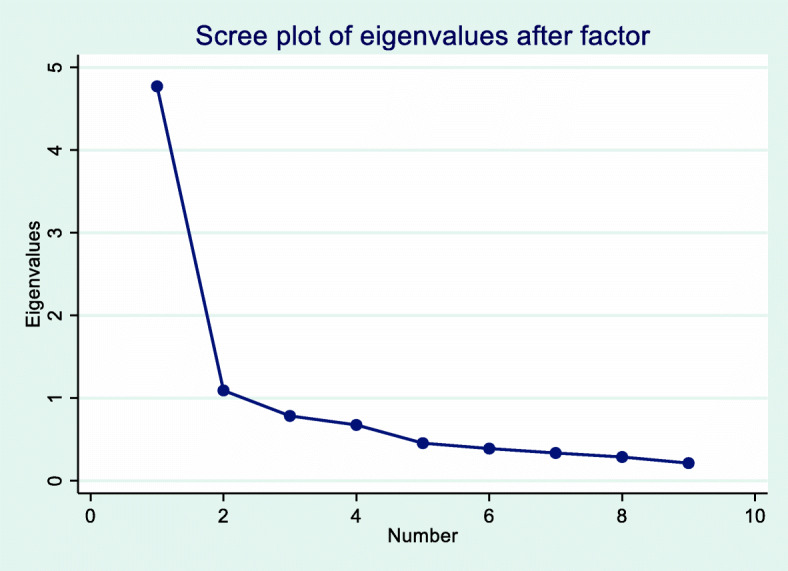
Test characteristic curve; y-axis: expected sum-score; x-axis = estimated Theta values, with a mean = 0 and a SD = 1, 95% of all patients with NSLBP have a Theta value between − 1.96 to 1.96 or between 0.984 to 20.7 in the FreBAQ sum-score

After fitting the GR IRT model, test characteristics were examined. The test characteristic curve (Fig. 2) shows the expected overall observed sum-score of the FreBAQ-G at each given θ-value of the underlying trait (back specific self-perception), which is plotted on the x-axis. It can be seen that the test characteristic curve is an increasing, nonlinear function of the underlying trait. This was anticipated because subjects with a higher perceptual impairment (right side of the x-axis) will be expected to score higher on the FreBAQ-G sum score. The test characteristic curve shows that 95% of the people with NSCLBP will score between 1 and 21 on the classical FreBAQ-G sum-score. In people with NSCLBP, who have an average self-perception, the sum-score will be 7, whereas a person with a self-perception 1 SD worse than average will reach a value of 14, and if the self-perception is 1 SD better above average, the score will drop to 2. Keeping in mind that higher values are an expression of more impairment, the figure also shows that the questionnaire can better differentiate people with a higher degree of impaired back specific self-perception, whereas people with a lower degree of perceptual impairment, the questionnaire offers only 7 points for discrimination (between zero points and seven points).

**Fig. 3:**
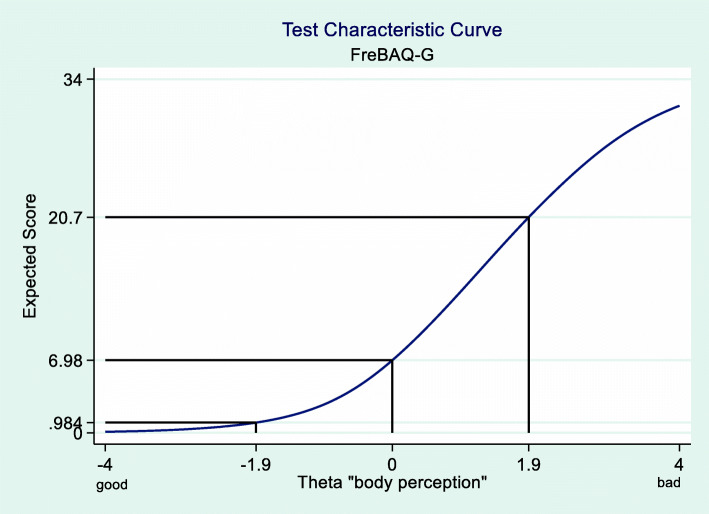
Test information function and standard error; Y-axis left side = test information; y-axis right side = standard error; x-axis = estimated Theta values

The test information function curve (Fig. 3) confirms that the FreBAQ-G is most informative for persons with a self-perception of the back worse than average (Theta between 0 and + 4).

The graph also shows that for persons with a self-perception of the back worse than the average, the SE (standard error of measurement) is lower than for those with a better than average self-perception of the back. The overall reliability of the FreBAQ-G in this study is 0.84. The reliability for self-perception of the back better than average is 0.797 and 0.884 for worse than the average.

### Item characteristics of the FreBAQ-G

Table 3 gives an overview of the item characteristics for the FreBAQ-G. All discrimination values of the nine items are above the desired value of 1. The highest discrimination parameter showed item 4, the lowest item 9. Because item discrimination and item information are related concepts it can be expected that item 4 offers the highest information whereas item 9 offers the lowest information about individual back specific self-perception. Table 3 also gives the item difficulty parameters for each item and category. The interpretation of each of the four item difficulty parameters per item is that a person equal to it has a probability of 50% for responding in the pertinent category or higher. For example, looking at the estimate of 0.87 for item 1 (“*My back feels as though it is not part of the rest of my body”)* category ≥2 means that a person with a back specific self-perception (Theta value) of that value (that is 0.87) has a probability of 50% to answer with category 0 or 1 versus response category 2 or higher (being 2, 3 or 4). Similarly, someone with a Theta value of − 0.07 has the same probability to answer 0 as to answer 1 or higher.

**Table 3:** Item characteristics FreBAQ-G

Nr	Item	Item dicrimination (^a^)	Item difficulty (^b^)				Info Rank
			≥ 1	≥ 2	≥ 3	= 4	
1	My back feels as though it is not part of the rest of my body	1.57	− 0.07	0.87	2.16	3.91	5
2	I need to focus all my attention on my back to make it move the way I want it to	1.39	−0.89	0.30	1.57	3.15	3
3	I feel as if my back sometimes moves involuntarily, without my control	1.85	−0.09	1.06	2.16	3.91	6
4	When performing everyday tasks, I don’t know how much my back is moving	2.31	−0.26	0.66	1.44	2.80	9
5	When performing everyday tasks, I am not sure exactly what position my back is	2.25	−0.20	0.60	1.35	2.47	8
6	I can’t perceive the exactly outline of my back	2.17	0.34	1.30	1.82	2.70	7
7	My back feels like it is enlarged (swollen)	1.41	0.85	1.66	2.74	–	4
8	My back feels like it has shrunk	1.30	1.35	2.38	3.66	–	2
9	My back feels lopsided (asymmetrical)	1.03	−1.26	−0.10	1.24	3.13	1

Item discrimination parameter (^a^) = higher values means better discrimination; item difficulty parameter (^b^) = higher values indicate more impairment, categories with higher values are easier; Info rank = higher values indicate more information about back specific self-perception.

**Fig. 4:**
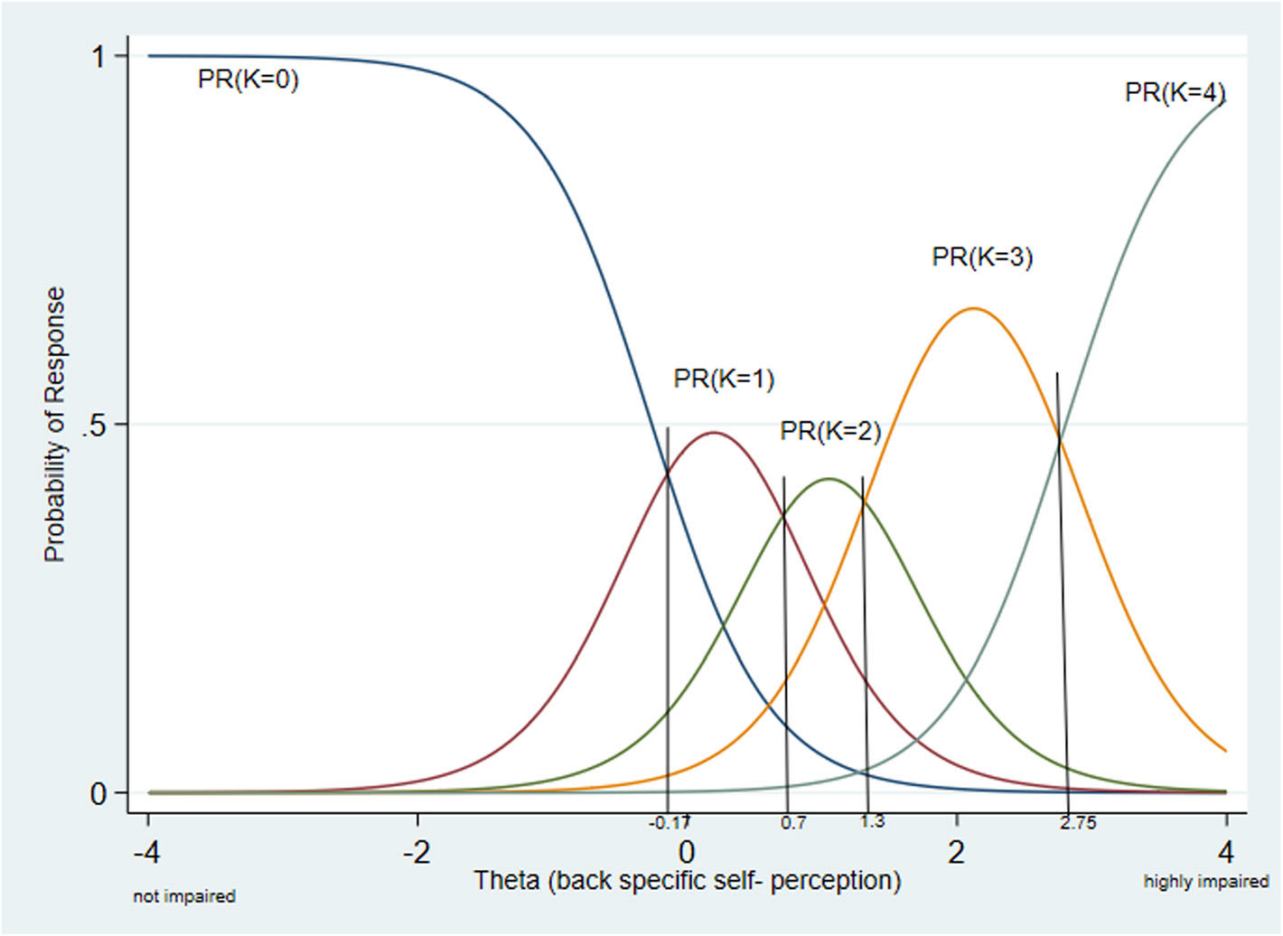
Category characteristic curve of item 4 showing the probability of highest response. y-axis: probability of response option; x-axis: estimated Theta values; Because of ordered responses the curves are arranged from category zero (K = 0, left side) to category 4 (K = 4, right side). To ease interpretation vertical lines at the crossing points of the response categories were added. A respondent with a Theta lower than − 0.17 is likely to respond category 0, a respondent with a Theta between − 0.17 to 0.7 is likely to respond category 1 and so on

Figure 4 shows the category characteristic curve of item 4 (“*When performing everyday tasks, I don’t know how much my back is moving”*). It can be seen that only the first and last response categories are monotonically decreasing and increasing. Furthermore, the categories can be considered as ordinal measures, so if one moves from left (not impaired) to the right (highly impaired) on the x-axis the probability of the response to the question of item 4 changes from category 0 (never feels like that) to category 4 (always, or most of the time feels like that). The crossing points can be described as transitions points. For instance, a Theta value of − 0.17 indicates a person whose back self-perception is 0.17 SD better than the average. In addition, for a person with a Theta value > − 0.17 the probability of choosing category 1 (rarely feels like that) becomes for the first time higher than choosing the reference category 0 (never feels like that). It is important to notice that each category has an interval where the probability for this response category is highest and that the intervals between the response categories are reflecting the ordinal structure of the questionnaire.

**Fig. 5:**
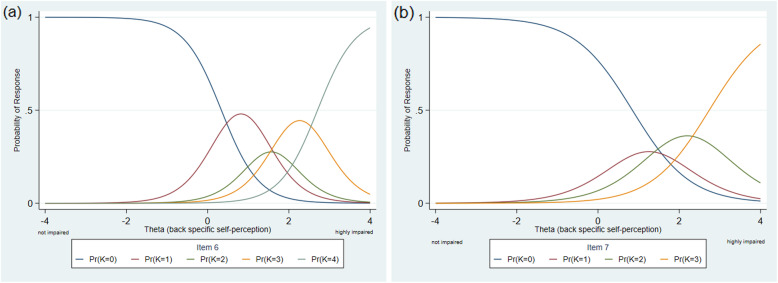
Category characteristic curve of item 6 (**a**) and item 7 (**b**). The response categories of item 6 and 7 are not properly ordered (compare to Fig. 4). For item 7 there is no region on the Theta-scale where the probability of choosing category 1 is higher than for the other categories. For item 6 this can be observed for response category 2

**Fig. 6:**
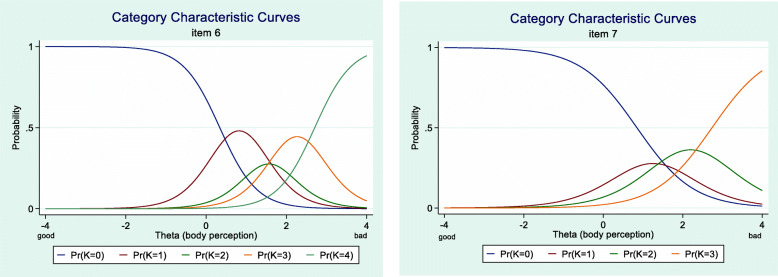
Calibrated mean item difficulty parameters for the German (y-axis) and the English (x-axis) language version. Scales on x- and y-axis represent item difficulty values and Theta values. All mean item difficulties are between 0 and 4 therefore the range of Theta values is only between 0 and 4. Line of identity with a slope of 1, dotted lines indicate the 95% CI boundaries

Examination of all category characteristic curves showed that for item 6 category 2 and for item 7 category 1 did not have a clear interval in the latent trait (see Fig. 5). Furthermore, none of the participants in the current German sample had sufficiently disrupted self-perception to choose category 4 (always, or most of the time feels like that) for items 7 and 8. The response categories of all items are shifted to the right side of the x-axis (e.g. see Fig. 6), except the categories of item 9 who are more equally distributed (see also Table 3).

### Differential item functioning of the FreBAQ-G

Only item 8 shows a small degree of differential item function (DIF) indicating that gender may influence the responses as for item 8 the item discrimination is lower in females (male 1.95; female 1.09), and item difficulty parameters are shifted to the right (thresholds: female: 1.48, 1.72, 4.16; male: 0.9, 1.63, and 2.5). However, these differences are not significant (*p* = 0.51). For all other items and subgroups we found no DIF in the German sample.

### Cross cultural validity

The calibrated mean item difficulties were plotted with the German items on the y-axis and the English items on the x-axis (Fig. 6). All difficulty parameters, except for item 7, lie within the 95% CI borders indicating equivalence of the item difficulty parameters of the English and German-language versions.

**Fig. 7:**
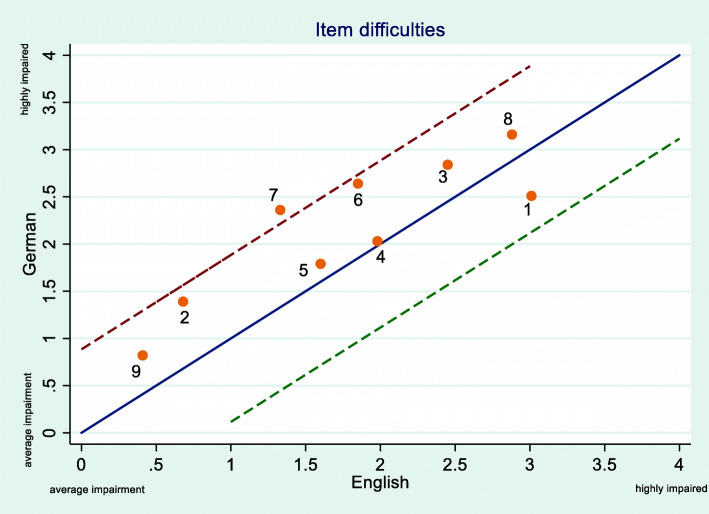
Item discrimination parameters for the German (y-axis) and the English (x-axis) language versions. Scales on x- and y-axis represent discrimination values. Blue line: line of identity with a slope of 1, dotted lines represent 95% CI. Higher item discrimination values are indicating better discrimination and higher information. Attention: the y and x-axis are not representing Theta values

Item discrimination parameters (a) of both populations were plotted in a similar way with the line of identity and 95% CI (Fig. 7). Again, all items lies within the 95% CI borders, indicating equivalence of the English and German versions. Values higher than 1 are desirable and indicate better discrimination. Items 4, 5 and 6 have the highest discrimination parameter in both populations.

**Fig. 8:**
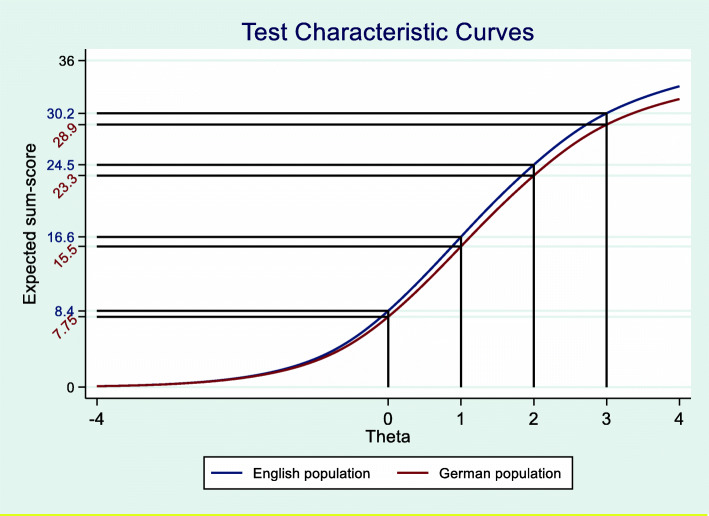
Test characteristic curve for the English and German-language population. Vertical and horizontal lines are displaying the expected sum-score for Theta values 0, 1, 2, 3 and 4

After accounting for the uniform differential item function (DIF) of Item 7 the graded response model (GRM) estimation of both groups showed a mean Theta of 0 with a variance of 1 for the English-language population (anchor) compared to a mean of − 0.1006 with a variance of 0.8348 for the German-language population. The test characteristic curve (Fig. 8) shows that individuals in the German population with a back specific self-perception below-average (Theta between 0 and + 4) will score one point lower on the FreBAQ than individuals with the same level of body perception in the English-language population. For individuals with above-average body perception (Theta 0 to − 4) there is no obvious difference in the expected sum-score.

### Correlation of back pain related parameters with the FreBAQ-G

Self-perception of the back showed significant, moderate correlations with pain intensity, disability and fear avoidance beliefs (Table 4).

**Table 4:** Correlations between the FreBAQ-G and pain related parameters

	Theta FreBAQ	Pain intensity	Pain disability	FABQ (sum score)	FABQ (activity)	FABQ (work)
**Theta** **FreBAQ**	1					
**Pain intensity**	0.421	1				
**Pain disability**	0.402	0.689	1			
**FABQ** **Sum score**	0.42	0.365	0.347	1		
**FABQ** **activity**	0.334	0.316	0.329	0.695	1	
**FABQ** **work**	0.346	0.284	0.25	0.887	0.286	1

*FABQ* sum-score of the Fear avoidance beliefs questionaire; all correlations are sig *p* < 0.001.

Multiple linear regression with the FreBAQ-G as the dependent variable showed that the best prediction model includes pain intensity, pain related disability and the sum-score of the FABQ (R-squared 0.27, *p* < 0.001). Eta square values indicate that the FABQ has the highest influence (0.092) and pain related disability the lowest (0.029). Other variables (e.g. demographic data) had no significant influence on the FreBAQ-G.

## Discussion

The primary aim of this study was evaluate psychometric properties of the FreBAQ-G using IRT in a large sample of patients with NSCLBP. Our results indicate that the FreBAQ-G is a suitable questionnaire to measure impaired back specific self-perception, and has comparable properties to the English-language version [20] Cross-cultural validation indicates that the English and the German-language versions are equivalent.

The FreBAQ-G showed good internal consistency (Cronbachs alpha 0.84), a good overall reliability (r = 0.84) and weak to moderate scalability (Loevinger Hj between 0.34 and 0.48). The questionnaire demonstrated unidimensional properties with factor loadings between 0.57 and 0.80 and at least moderate correlations (r > 0.35) with pain intensity, pain related disability and FABQ total - and subscores.

The participant characteristics in German-language study were comparable to those in the English-language study [20], except that our participants were 6 years younger, had 2 points less average pain intensity (0–10 NRS), and 4 points less fear avoidance (FABQ-PA) on average. The correlations of FreBAQ scores with average pain, disability and FABQ-PA reported in for the English FreBAQ were comparable to the present study, with correlation coefficients ranging between 0.33 and 0.42 [20].

### Frequencies and responses for each FreBAQ-G item

The average total FreBAQ-G score in our sample was slightly lower with an average of 8.0 (SD 6.0) compared to Wand et al. [20] with 9.8 (SD 6.6). This difference can be explained by the slightly lower pain intensity and FABQ-PA level of our sample, which may also be a reason for the observed floor effect of the sum-scores (compare to Fig. 1). For items 7 and 8, none of the participants in the German-language population scored category 4 (always, or most of the time feel like that). In line with Wand et al. [20], item 9 had the lowest mean difficulty parameter and item 8 the highest.

### Internal consistency, reliability and homogeneity of the FreBAQ-G

The calculated reliability index of our study was r = 0.84 (range 0.79–0.88) and Cronbach’s Alpha was 0.84. These values are comparable to those found by Wand et al. [20] (CA = 0.80, r = 0.74). Loevinger Hj coefficients, referring to the questionnaires ability to differentiate persons into score groups [29], showed moderate scalability for all items except for items 8 and 9. Here the values were slightly lower (below 0.4).

### Structure of the FreBAQ-G

In line with Wand et al.’s study, we found that the scale of the FreBAQ-G reflects an unidimensional construct. Compared to Wand et al. [20], the PCA in our study showed more robust results. The eigenvalue of the first factor was greater than 4 and explained 53% of the variance, compared to Wand et al. [20] with an eigenvalue greater than 2. Based on X^2^ statistics the assumption of local independence could not be rejected. The use of the IRT-graded response model (GRM) was supported by the model-fit and item-fit of the data.

### Test properties of the FreBAQ-G

The FreBAQ-G is suitable to differentiate between people with back specific self-perception between Theta − 1 and + 2 (− 4 stands for not impaired and + 4 stands for highly impaired) (see also Fig. 3). For people with a better back specific self-perception perception (Theta values below − 1), other items have to be developed. This view is supported when looking at the FreBAQ-G sum-score that ranges from 0 to 45. People with an average impairment of self-perception of the back had a median score of 8. This means that for patients with worse than-average self-perception the score ranges from 8 to 45 whereas for patients better than-average, the available score range is only from 0 to 8.

Furthermore, the classical sum-score of the FreBAQ-G should be used with care. The sum-score can only have integer values, and because of the non-linear relationship between the sum-score and the theta score, the assumption of equal distances between scores is violated. Therefore the sum-score is not interval scaled and even its ordinal scale can be questioned.

### Cross cultural validity

We used the graded response model (GRM) to compare the performance of both language versions. The English-language version showed very good model fit for the graded response model (GRM). All items showed comparable item discrimination parameters. In both populations items 4, 5 and 6 showed the highest discrimination. Therefore, in both language versions, the responses to these three items are offering the highest amount of information about the back specific self-perception of the respondent. Only in the English-language version Items 8 and 1 had discrimination parameters lower than 1. We found no non-uniform differential item function (DIF). However we found uniform differential item function (DIF) in item 7. This does not automatically indicate that translation of these items was not accurate. Differential item function (DIF) can also occur due to chance, different sample sizes, group differences in age, sex or disease characteristics, different administration modes and real differences between cultural and language settings [31]. As our total sample size was large and evenly distributed between the two language groups, differential item function (DIF) is unlikely to be due to chance or sample size differences. Differences in age, sex, or disease characteristics between the German and the English-language group are more likely explanations. There are differences between the two language groups in terms of age, BMI, pain intensity and the FABQ. The German language group was significantly younger, had a lower BMI, lower pain intensity and a lower mean score in the FABQ. Figure 6 shows that for eight items the calibrated mean item difficulty parameter lies above the line of equality in the German-language population. We think that this is a sign of a systematic difference between these two study populations. Therefore we believe that the differential item function (DIF) observed in item 7 as well as the other observed differences in the performance of the FreBAQ between these two populations were due to the described sample differences.

### Limitations

Some limitations of our study need to be discussed. First of all, the sample size is at the lower margin of sample sizes recommended for IRT studies. However, our results are sufficiently precise to assume that a larger sample size would not alter their magnitude or direction. Also, levels of pain intensity were relatively low in comparison to Wand et al’s study [20], therefore comparability between results is compromised.

### Clinical implications

Our results indicate that the FreBAQ-G gives the most valid results for persons with NSCLBP and physical impairment above average. The item discrimination values of item 4, 5 and 6 show that these provide the most information in regards to impaired back specific self-perception.

For clinical interpretation of FreBAQ-G sum-scores, a common metric is very helpful, especially when the results of different measurement instruments have to be compared [36]. However, the theta-scale has negative and positive values and it might be difficult to communicate their meaning. To aid the interpretation of the individual trait level estimates we recommend a T-transformation. The T-transformation is defined as follows:

$$ \mathsf{T}=\mathsf{50}+\mathsf{10}\ \mathsf{x}\ \mathsf{Theta} $$

T-scores have a range from 0 to 100 with a mean of 50 and a SD of 10. Higher values indicate more pronounced perceptual impairment of the back. T-scores are interval-scaled, easy to interpret and T-scores from different questionnaires can easily be compared. E.g. if a person with NSCLBP has a T-score of 65 we know that their body perception is 1.5 SDs worse than average, or if another person has a T-score of 40 the body perception is 1 SD better than average. Assessment of impaired self-perception could add information to the complex clinical picture of NSCLBP and thereby help clinicians to select targeted treatment options.

### Implications for future research

Future longitudinal studies should investigate sensitivity to change of the FreBAQ. It would add confidence to the validity of the questionnaire, if improvement in outcomes such as pain, function or health-related quality of life correspond to changes in self-perception of the back. Furthermore, the FreBAQ could be used as an outcome measure in controlled trials investigating interventions that target impaired self-perception of the lower back, such as tactile discrimination training or visual feedback training, results may help to explore how treatments work or why treatments do not work.

## Conclusions

The FreBAQ-G is best suited for determining impaired back specific self-perception in patients with NSCLBP who have worse than average self-perception. Our results indicate cross-cultural equivalence that is important for the comparison of international study results. We found positive correlations of impaired back self-perception to pain intensity, disability and fear avoidance beliefs, in line with previous findings.

## Supplementary Information

**Additional file 1: Appendix** Supplementary statistical analysis

## Data Availability

The datasets during and/or analysed during the current study are available from the corresponding author on reasonable request.

## Abbreviations

df: Degree of freedom
FABQ: Fear avoidance and Beliefs Questionnaire
FreBAQ-G: Fremantle back awareness questionnaire German version
IRT: Item Response Theory
LBP: Low back pain
NSCLBP: Non-specific chronic low back pain
NRS: Numerical rating scale
PCA: Principal Component Analysis
RMSEA: Root mean square error of approximation
SD: Standard Deviation
SE: Standard error
