# Supplementary material for: Validation and investigation of cross cultural equivalence of the Fremantle back awareness questionnaire - German version (FreBAQ-G)
Source: BMC Musculoskelet Disord. 2021 Apr 2;22:323. doi: 10.1186/s12891-021-04156-1 (PMC8017790; doi:10.1186/s12891-021-04156-1)
Supplement: Supplementary file 1 — Additional file 1: Appendix Supplementary statistical analysis [file 12891_2021_4156_MOESM1_ESM.docx]

**Appendix: Supplementary statistical analysis**

**Validation and investigation of cross cultural equivalence of the Fremantle Back Awareness Questionnaire - German version (FreBAQ-G)**

**Authors**

Axel Schäfer, Benedict Wand, Kerstin Lüdtke, Katja Ehrenbrusthoff, Thomas Schöttker-Königer

1. **Test of IRT assumptions: dimensionality and local independence:**

Local independence and unidimensionality are two connected and important assumptions in IRT modelling. Unidimensionality refers to the aspect that all items of a questionnaire are measuring the same latent trait [1, 2]. In the case of the FreBAQ-G, all items should measure a component of “self-perception” of the low back [3]. To meet the assumption of local independence responses to items of the questionnaire should be uncorrelated when the measured ability (back specific self-perception) is fixed. Unidimensionality was statistically tested using Principal Component Analysis (PCA). Given the ordinal nature of the item response categories a PCA based on polychoric correlation matrix was used. Unidimensionality was considered to be present if the first factor had an eigenvalue >1 and accounts for at least 20% of the variability. In addition, the ratio of the variance explained by the first to the second factor should be > 4 and the factor loadings of all items should be > 0.5 [4].

To assess local independence, the standardized linkage disequilibrium *X*^2^ statistic [5] for each item pair was examined. linkage disequilibrium X^2^ values >10 were considered to reflect a violation of the local independence assumption; values between 5 and 10 reflect questionable local independence, and values < 5 were considered as adequate to accept the local independence assumption [6]. Item level fit for each item was assessed using S-X^2^ statistic. This statistic assesses the degree of similarity between the predicted model and the observed response frequencies by item response category [6]. A statistically significant p-value indicates that the model does not fit a given item. Finally, model level fit was assessed using the root mean square error of approximation (RMSEA). The RMSEA ranges from 0 to 1 with values closer to zero indicating adequate model-data fit. RMSEA values < 0.08 are indicating good fit [7].

**Results**

The PCA _polychoric_ showed that the eigenvalue of the first factor is 4.77, explaining 53% of the variance (Figure 2). The ratio of the variance explained by the first to the second factor was 4.41. The loading of the items was between 0.57 (item 9) and 0.8 (item 6). Unidimensionality of the scale is therefore assumed.

**
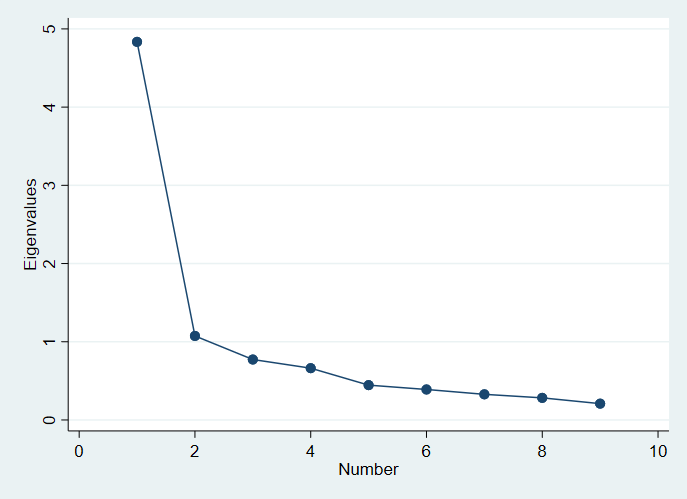
**

**Figure 1**: Scree plot of the FreBAQ-G based on PCA _polychoric_

Examination of the 9x9 marginal fit (X^2^) and standardized linkage disequilibrium X^2^ matrix shows that the values range between 0 and 3.4 with only two values > 3 and 5 values > 2. Therefore, the assumption of local independence can be accepted. The S-X^2^ item level diagnostic shows that p-values range from 0.1247 (item 9) to 0.6431 (item 3), indicating sufficient item level fit. The RMSEA of 0.04 indicates good model-data fit.

1. **Test of IRT model:**

As proposed by Raykov and Marcoulides [1], we used the Akaike information criterion (AIC), the Bayesian information criterion (BIC) and the likelihood-ratio test to select the best fitting IRT model.

***Result****:*

Based on Akaike information criterion (AIC) and Bayesian information criterion (Bayesian information criterion (BIC)) the graded response model (graded response model (GRM)) best reflects the data (Table 2). The likelihood-ratio test (likelihood ratio test) supports this choice and shows that the difference between the graded response model (GRM) and the partial credit model (PCM) is significant (LR Chi^2^(8) 54.26, p<0.001). The graded response model (GRM) is appropriate for ordered polytomous responses such as Likert scales [1]. The graded response model (GRM) partitions the number of items into blocks (thresholds). The thresholds can be defined as the level at which a likelihood of a response category below the threshold turns to a likelihood of success [8]. A five category Likert scale has four threshold parameters. In contrast to the RSM, the graded response model (GRM) calculates different discrimination factors for each item.

**Table 1:** IRT models and goodness of fit

| Modell | N | ll(model) | df | Akaike information criterion (AIC) | Bayesian information criterion (Bayesian information criterion (BIC)) |
| --- | --- | --- | --- | --- | --- |
| graded response model (graded response model (GRM)) | 271 | -2571.179 | 43 | 5228.359 | 5383.25 |
| partial credit model (PCM) | 271 | -259.949 | 35 | 5265.898 | 5391.972 |
| generalized partial credit model (GPCM) | 271 | -2579.396 | 43 | 5244.793 | 5399.684 |
| rating scale model (RSM) | Because some items have 4 and some 5 levels rsm can not be used | | | |  |

Akaike information criterion (AIC)=Akaike information criterion;Bayesian information criterion (Bayesian information criterion (BIC))= the Bayesian information criterion; ll=LogLikelihood; df=degree of freedom; N=sample size

**Literature**

1. Raykov T, Marcoulides GA: **A Course in Item Response Theory and Modeling with Stata**. Texas: Stata Press; 2018.

2. DeVet H, Terwee CB, Mokkink LB, Knol DL: **Measurement in Medicine**: Cambridge University Press; 2011.

3. Wand BM, Catley MJ, Rabey MI, O'Sullivan PB, O'Connell NE, Smith AJ: **Disrupted Self-Perception in People With Chronic Low Back Pain. Further Evaluation of the Fremantle Back Awareness Questionnaire**. *J Pain* 2016, **17**(9):1001-1012.

4. Reeve BB, Hays RD, Bjorner JB, Cook KF, Crane PK, Teresi JA, Thissen D, Revicki DA, Weiss DJ, Hambleton RK *et al*: **Psychometric Evaluation and Calibration of Health-Related Quality of Life Item Banks: Plans for the Patient-Reported Outcomes Measurement Information System (PROMIS)**. *Medical Care* 2007, **45**(5):S22-S31.

5. Liu Y, Thissen D: **Identifying Local Dependence With a Score Test Statistic Based on the Bifactor Logistic Model**. *Applied Psychological Measurement* 2012, **36**(8):670-688.

6. Toland MD: **Practical Guide to Conducting an Item Response Theory Analysis**. *The Journal of Early Adolescence* 2013, **34**(1):120-151.

7. Hooper D, Coughlan J, Mullen M: **Structural Equation Modeling: Guidelines for Determining Model Fit**. *The Electronic Journal of Business Research Methods* 2007, **6**.

8. Raju D, Su X, Patrician PA: **Using item response theory models to evaluate the Practice Environment Scale**. *Journal of nursing measurement* 2014, **22**(2):323-341.
